# Exercise to preserve beta cell function in recent-onset type 1 diabetes mellitus (EXTOD) - a study protocol for a pilot randomized controlled trial

**DOI:** 10.1186/1745-6215-14-180

**Published:** 2013-06-18

**Authors:** Nadia Lascar, Amy Kennedy, Nikki Jackson, Amanda Daley, George Dowswell, Dylan Thompson, Keith Stokes, Sheila Greenfield, Roger Holder, Rob Andrews, Parth Narendran

**Affiliations:** 1 Clinical and Experimental Medicine, University of Birmingham, Birmingham, UK; 2 Division of Medicine, University of Bristol, Bristol, UK; 3 Primary Care Clinical Sciences, University of Birmingham, Birmingham, UK; 4 Sport and Exercise Science, University of Bath, Bath, UK; 5 School of Clinical Sciences, University of Bristol, Bristol, UK; 6 School of Clinical and Experimental Medicine, College of Medical and Dental Sciences, Institute of Biomedical Research, University of Birmingham, Edgbaston, B15 2TT, Birmingham, UK

**Keywords:** Exercise, Type 1 diabetes, Beta cell function, Physical activity, Barriers, Lifestyle, C peptide

## Abstract

**Background:**

Exercise has a beta cell preserving effect in patients with type 2 diabetes. This benefit of exercise has not been examined in type 1 diabetes. Significant beta cell function is present at the time of diagnosis of type 1 diabetes and therefore studies of beta cell preservation are ideally conducted immediately after diagnosis.

Many of the variables required to design and power such a study are currently unknown. The aim of EXTOD is to obtain the information required to design a formal study of exercise and beta cell preservation in newly diagnosed patients with type 1 diabetes.

**Methods:**

Barriers to exercise will initially be assessed in a qualitative study of newly diagnosed patients. Then, sixty newly diagnosed adult type 1 diabetes patients will be randomized to either conventional treatment or exercise, stratified on beta cell function and fitness. The exercise group will be encouraged to increase their level of activity to a minimum of 150 minutes of moderate to vigorous intensity exercise per week, aiming for 240 minutes per week of exercise for 12 months. Beta cell function will be measured by meal-stimulated C peptide. Primary outcomes are recruitment, adherence to exercise, loss to follow-up, and exercise levels in the non-intervention arm (contamination). The secondary outcome of the study is rate of loss of beta cell function.

**Discussion:**

The outcomes of the EXTOD study will help define the barriers, uptake and benefits of exercise in adults newly diagnosed with type 1 diabetes. This information will enable design of a formal study to assess the effect of exercise on beta cell preservation in newly diagnosed patients with type 1 diabetes.

**Trial registration:**

Current controlled trials ISRCTN91388505

## Background

### The natural history of beta cell loss in type 1 diabetes

Type 1 diabetes (T1DM) is a chronic inflammatory autoimmune disease characterized by destruction of insulin producing beta cells and by subsequent insulin deficiency [1]. It affects 0.3% of the UK population, approximately 250,000 people, and its incidence is rising [2]. In the UK, it has been reported to result in a shortening of life expectancy by over 20 years [3].

The loss of beta cells that results in T1DM is a gradual process, and between 50 and 25% of beta cell function can be present at the time of diagnosis [4]. However this residual function is insufficient to generate the insulin required for metabolic control, and the lack of insulin results in a number of metabolic derangements which if untreated results in death. Patients with T1DM therefore start insulin injection replacement therapy at diagnosis. It has generally been assumed that the remaining beta cells are rapidly and completely destroyed soon after diagnosis. However, recent studies indicate that these cells can persist for over 50 years following diagnosis [5-7], and that their presence is associated with important clinical benefits. These benefits include improved glucose control, reduced retinopathy and nephropathy, and with more than a halving of rates of hypoglycemia [8-11]. These benefits of preservation of beta cell function are significant, and the FDA (USA), and EMEA (Europe) state that even partial preservation of beta cell function ((estimated by a stimulated C peptide > 200 pmol/L - elaborated below)) is a sufficient basis for the licensing of new therapies for T1DM [12].

### What are the benefits of beta cell preservation in people with type 1 diabetes?

Whilst beta cell mass cannot be directly measured, it can be accurately estimated through measurement of stimulated C peptide (a component of the pre-insulin molecule) following a physiological meal stimulus [13]. Meal-stimulated C peptide provides an acceptably accurate estimate of beta cell function for use in studies of beta cell preservation [14]. A meal-stimulated C peptide value of greater than 200 pmol/L is associated with the clinical benefits outlined above. C peptide assay technology has improved over the recent years, and through National Institute of Diabetes and Digestive and Kidney Diseases (NIDDK) sponsored standardization workshops, levels of 30 pmol/L can now be measured reliably and reproducibly across different laboratories [15]. More recently, the development of sensitive assays has allowed C peptide detection down to levels of 1.5 pmol/L [16]. Such assays have provided even stronger support for the persistence of beta cell function in people with long standing T1DM, albeit at levels that do not provide independence from insulin injections [17]. Current estimates therefore are that 60% of subjects will have a stimulated C peptide greater than 200 pmol/L at two years following diagnosis, but that C peptide is detectable at lower levels in 64% of people at 50 years after diagnosis [7].

### How can we preserve beta cell function in people with type 1 diabetes?

A number of medicinal products are currently under investigation for the preservation of beta cell function in subjects newly diagnosed with T1DM. They are largely immunomodulatory agents that act by ‘suppressing’ the inflammatory autoimmune process targeting the beta cell. Whilst some of them act to modulate the autoimmune process specifically against beta cell antigens [18], others act through broad, non-antigen specific immune suppression [19]. Many of these therapies are associated with significant risk of side effects. These include infection or reactivation of latent infection, inflammation at the sites of injection, and the risk of cancer and infection associated with immunosuppressive therapy [20]. Furthermore, these new therapies have yet to demonstrate significant and sustained benefit. Whilst we clearly need to continue investigating such novel therapies, there is also a pressing need to examine new therapies with an acceptable side effect profile, and which potentially could be used as an adjunct to the medicinal products under investigation. We are interested in the role of exercise in this regard.

### Can exercise preserve beta cell function in people with type 1 diabetes?

Exercise has been demonstrated to preserve beta cells in animal studies [21-23]. Pancreatic sections from a rat model of insulin deficient diabetes revealed a 33% increase in staining for beta cells, and a 31% increase in beta cell mass following an eight-week exercise program. The mechanisms underlying this effect remain unclear but a significant decease in beta cell apoptosis was reported [24]. Exercise has a recognized anti-inflammatory effect [25] that may therefore modulate the autoimmune process against the islet.

These beneficial effects of exercise on beta cell function have also been demonstrated in healthy human subjects [26], and in the context of people with pre- or established type 2 diabetes [27,28]. Here also, the exercise has been of vigorous intensity with a VO2max of 70%, and of sustained duration. The disposition index used in this study as an estimate of beta cell function, revealed a 27% improvement following a one-week program of exercise in older people with impaired glucose tolerance [27]. More recently, an eight-month exercise program in middle-aged overweight people revealed a 60% improvement in beta cell function and a 20% improvement in insulin resistance with moderate intensity exercise (walking at a slow pace for 60 minutes on three days per week) [29].

Until recently, many observers have remained skeptical that increases in exercise can be maintained long enough to have any significant impact on diabetes risk or help improve diabetes management. However, large intervention studies targeting subjects with impaired glucose tolerance have demonstrated that a program of lifestyle changes focusing on improved diet and increased exercise is able to delay or possibly prevent the development of type 2 diabetes mellitus (T2DM) over a period of four years [30,31]. Both studies found lifestyle intervention to result in a 58% reduction in the incidence of diabetes, irrespective of age. Importantly, both studies also showed that it was possible to maintain increased levels of exercise for four years. Furthermore, we ourselves have shown that a program of lifestyle changes focusing on improved diet and increased exercise, reduces insulin resistance, weight, and drug usage and improves diabetes control in newly diagnosed type 2 patients [32]. In this study, we demonstrated that with a simple home-based, ‘unsupervised’ and relatively inexpensive exercise regime we can maintain increased levels of exercise for 12 months.

We therefore believe that regular exercise, if demonstrated to show benefit in patients with residual beta cell function, can be undertaken and maintained by patients with diabetes.

### Hypothesis

Our hypothesis is that exercise preserves beta cell function in people with T1DM. We base this hypothesis on previous studies of exercise in T2DM, pre-T2DM, and people without diabetes, where exercise is associated with preservation of beta cell function.

### Research goals

In order to test our hypothesis, we will initially undertake a qualitative study to identify barriers to the uptake and adherence to an intensive exercise program, and determine the most acceptable way to monitor exercise levels. We will then undertake a pilot RCT involving an exercise intervention in patients with recent onset T1DM in order to:

1) Determine the proportion and characteristics of patients with T1DM who would be willing to take part in an RCT of exercise (that is, recruitment rate).

2) Define the rates of exercise adherence to the intervention and participant drop-out. The unsupervised exercise program will aim to encourage patients to safely increase and maintain their exercise level to at least 150 minutes per week, and aiming for 240 minutes per week of vigorous intensity exercise. A minimum level of 150 minutes per week is recommended by major diabetes organizations [33,34]. However, to maximize the chances of seeing a benefit in beta cell function, patients will be encouraged to aim for 240 minutes per week.

3) Determine the rate of exercise uptake in the non-intervention arm (that is, intervention contamination). This issue is important because it has the potential to dilute trial effects, and adjustments for this will need to be made when calculating the sample size for a definitive trial.

4) Determine the rate of loss of beta cell function (potential effect size) in the intervention and control arm to enable the statistical power calculations for the subsequent definitive trial to be refined.

5) Determine (as a secondary outcome) whether the 12 months exercise intervention results in a significant preservation of beta cell function.

## Methods and design

### Setting and recruitment

The study will take place across 19 NHS hospital Trusts in the UK: University Hospital Birmingham, Taunton and Somerset, University Hospitals Bristol, North Bristol, Gloucester Hospitals, Yeovil District Hospitals, East and North Hertfordshire, Mid Yorkshire Hospitals, Oxford University Hospitals, Worcestershire Acute Hospitals, George Eliot Hospital, The Dudley Group, Walsall Healthcare, The Royal Wolverhampton Hospitals, Heart of England, Sandwell and West Birmingham, Weston Area Health, Royal United Hospital Bath and Royal Devon and Exeter Hospital. Recruitment will focus on NHS hospital trusts as the vast majority of patients newly diagnosed with T1DM are referred to hospital for initiation of insulin.

### Participant selection

Clinical staff at participating hospitals will identify patients newly diagnosed with T1DM. They will approach these patients about the study and ask permission for their contact details to be forwarded to the EXTOD study team. Interested participants will then be contacted by the study team by telephone and invited to attend their local study centre for a screening visit. At the first visit written informed consent will be obtained by a member of the study team (physician or research nurse). Patients will be considered eligible for enrollment in this trial if they fulfill all the inclusion criteria and none of the exclusion criteria (see Table 1).

**Table 1:** Inclusion and exclusion criteria

**Inclusion criteria**	Aged 16 to 60
	Diagnosed with T1DM within the previous 12 weeks
	Able and safe to exercise (as determined by the lead physician)
	Willing to self-monitor and record blood glucose levels
	Willing to take insulin as part of a multiple dose injection regime
	Feel able to increase their current levels of exercise
	Have a meal-stimulated C peptide value greater than 200 pmol/l (these criteria are not required for recruitment into phase 1)
**Exclusion criteria**	Psychological or physical disease that prevents exercise
	Concomitant therapy that affects heart rate (for example, beta blocker, calcium channel antagonist) as we would be unable to monitor their exercise adherence
	Major surgery or other planned event that would prevent exercise for more than six weeks
	Pregnancy or planning pregnancy
	Uncontrolled blood pressure (greater than 180/100 mmHg), as it is unsafe to exercise with this blood pressure

### Study design

The EXTOD study is designed in two phases. Phase 1 is a qualitative study designed to inform on the most feasible and patient-friendly way of motivating patients newly diagnosed with T1DM to undertake and maintain a graded exercise program, and to determine the most acceptable way to monitor exercise levels. This understanding is essential for the conduct of phase 2.

Phase 2 is a pilot RCT to assess uptake, intervention adherence, drop-out rates, and rate of uptake in the usual care group during a 12 month exercise intervention, and the effect of this intervention on beta cell function.

### Phase 1 - qualitative study

Patients will be interviewed once face-to-face by a qualitative researcher. The interview format will be flexible to allow for logistics of the large area being covered by the study and interviews will take place either on an individual basis or with two or more patients together as appropriate. Interviews will be semi-structured to ensure that key themes are covered, whilst giving participants the opportunity to freely express their views.

The topic guide will explore participants’ views and experience of type 1 diabetes, and what information they were given about exercise at diagnosis (Table 2). All interviews will be recorded, and interviewing will continue until saturation is reached.

**Table 2:** Topic guide for Phase 1 interviews

1) Moderator’s introduction	
2) About the group	
3) Activity and exercise behavior	What exercise do you do?
	What does the word ‘exercise’ mean to you? (focus on activity levels)
	Why should someone exercise?
	How active are you on a day to day basis/how do you feel about the amount of exercise you do?
	Do you think you do enough exercise to keep healthy?
	Is exercise important in the management of diabetes?
	What are the recommended guidelines?
	The DOH recommends 150 minutes exercise per week, what do you think of this?
	Do you think this is achievable?
	How do you relate to the recommended levels?
4) Barriers to exercise	What are the mains reasons for not meeting the guidelines?
	How do you try to overcome these barriers? How can you resolve them?
	If you have a fear of having a hypo, do you make any adjustments?
	If you had a magic wand what would be the one thing you could overcome in order to allow you to do more exercise?
	Has the diagnosis of diabetes changed your attitudes towards exercise?
	Does education and understanding have a role in the management of your diabetes and therefore your exercise levels?
5) Encouragement and facilitation of exercise	Can you think of any ways of improving your activity levels?
	How can small changes be incorporated into your lifestyle?
	Are there any major themes that would help encourage people to be more active?
	Would more advice or information help?
	If you had to choose one intervention that would help your activity levels - which would it be?
	Has anyone any successful experiences of exercising?
	We are thinking about doing a study - if you were to take part how would you like to be monitored / encouraged?
	One-to-one advice from a health and fitness advisor
	Attending an exercise group organized by the hospital or your GP
	Support - someone who keeps in touch to see how you are doing with your exercise program
	Goal setting/modification/action planning
	Heart rate monitoring
	Chat room with other people from the study to share ideas
	Uploading BMI/weight loss onto website - self monitoring
	If phone calls weren’t appropriate, what else could we do to motivate you?
6) Summary of session	Outlining main points of discussion and key issues raised
	Questions and thank everyone for their input

### Analysis

Data collection and analysis will be iterative to allow earlier data to shape later data collection. The transcribed data will be analyzed using constant comparative analysis to identify the emerging themes which comprise interviewees’ views regarding particular issues [35]. Themes from participants of different ages and gender will be compared and differences and similarities noted. Management of data for analysis will be assisted by the software package NVivo (QSR International, Victoria, Australia). Themes and a coding frame will be developed by reading and re-reading of the interview transcripts and through discussions between members of the multi-disciplinary research team.

Information from this phase will be used to refine the intervention developed for phase 2.

### Phase 2 - RCT

#### Pre-randomization visits

Patients will be initially screened on the telephone and potentially eligible individuals invited to attend a face-to-face assessment to confirm eligibility (visit 1). Here the study team will obtain informed consent, record their clinical history and conduct a physical examination. Participants will return in a fasting state to undertake baseline tests including the meal-stimulated C peptide measurement (visit 2, Table 3). Participants will return again to undertake Astrand-Rhyming and YMCA/ACSM cycle tests to assess fitness [36] (visit 3). Two cycle tests will be performed to ensure that we can get an accurate estimation of fitness. A study doctor will see all patients at visit 1.

**Table 3:** Tests/action/questionnaires conducted at baseline, 6 and 12 months

Clinical examination	Cardiovascular/respiratory/gastrointestinal/nervous system/feet
	Blood pressure and heart rate
	Height, weight, waist circumference
	Body-fat content (bio-impedance)
Non-fasting blood collection	GAD, IA-2 and zinc transporter autoantibodies
	Full blood count/thyroid function
	Liver/renal function
	DNA (optional, only at visit 1)
Fasting blood collection	Cholesterol, LDL, HDL, triglycerides
	Serum/plasma storage
Inflammation markers	Adiponectin, leptin, IL-6, IL-10, CRP
Meal-stimulated C peptide	Participant attends fasting and drinks Fortisip 240 ml. Venous blood samples collected at time -10, 0, 15, 30, 60, 90, 120 minutes
Questionnaires	International physical activity questionnaire (IPAQ)
	Bandura exercise self-efficacy questionnaire
	Social support for physical activity scale
	Outcome expectations for exercise
	Deci and Ryan motivational questions TSRQ
	Health care climate questionnaire HCCQ
	Pittsburgh sleep quality index (PSQI)
	WHO quality of life-BREF
	Fear of hypoglycemia survey
	BRIEF illness perception
	Problem areas in diabetes (PAID)
	CES-D
	Toole and Glasgow dietary questionnaire
	EQ-5D
Activity monitor	Small electronic device worn during waking hours for seven days. Measures physical activity by continually monitoring and recording movements of the body
Fitness test	Estimation of VO2max by submaximal test
Nurse visit	Education on carbohydrate counting and insulin dose adjustment around exercise
	Review of exercise diaries
	Hypoglycemia management
	Motivational support
Focus group for feedback	Obtain feedback on the study

#### Randomization

Randomization will be done according to computer-generated allocation. Patients will be assigned, in a 1:1 ratio, to ‘usual care’ or ‘exercise intervention’. Allocations will remain concealed by the trial coordinator until the patients attends visit 4 when they will be randomized using on-line software. Randomization will be minimized by center, meal-stimulated 90-minute C peptide, and fitness. Due to the nature of the intervention, blinding of the study team to the randomization arm is not possible.

#### Post-randomization visits

In agreement with the local GPs and hospital doctors, the study team will undertake the ongoing diabetes management of all trial participants for the period of the trial. This is to ensure that similar guidelines for treatment intervention are maintained across all sites. Patients will be seen at baseline, 6 and 12 months by a doctor and any changes in treatment will be made according to strict guidelines, thus ensuring no bias between the two groups. All participants will have eight visits. Those patients randomized to the exercise arm will have a further five telephone consultations (see Table 4).

**Table 4:** Visit overview

	**Pre-randomization**			**Randomization**	**Post-randomization**													
**Test/action**	**Visit 1**	**Visit 2**	**Visit 3**	**Visit 4 (wk 0)**	**Visit 5 (wk 2)**	**Visit 6 (wk 4)**	**Visit 7 (wk 8)**	**Visit 8 (wk 12)**	**Visit 9 (wk 16)**	**Visit 10 (wk 20)**	**Visit 11 (wk 24)**	**Visit 12 (wk 24)**	**Visit 13 (wk 30)**	**Visit 14 (wk 36)**	**Visit 15 (wk 42)**	**Visit 16 (wk 48)**	**Visit 17 (wk 48)**	**Visit 18 (wk 50)**
Clinical exam	x							x			x			x			x	
Questionnaires	x	x									x						x	
Activity monitor	x							x			x			x			x	
Non-fasting blood tests	x										x						x	
Fasting blood tests		x									x						x	
HbA1c	x							x			x			x			x	
Inflammatory markers		x									x						x	
Urine albumin/creatinine ratio		x									x						x	
Meal-stimulated C peptide		x									x						x	
Fitness test			x									x				x		
Nurse visit					x	X^a^	x	x	X^a^	x			X^a^	x	X^a^		X^a^	
Focus group feedback																		x

X: visit attended; wk: week. ^a^visit for exercise intervention arm only.

#### Usual care

Usual care will consist of standard dietary and exercise advice after randomization, and at the end of the study. There will also be reviews by a study doctor and nurse at baseline and at 6 and 12 months. At randomization, the dietitian will meet with the patients for 45 minutes to provide education on food intake as well as providing information on insulin and carbohydrate dose adjustment around exercise and hypoglycemia management. Any changes to medication will be made using a standardized staged protocol (see disease management).

#### Exercise intervention

Participants allocated this arm will receive dietary advice at baseline as for the usual care arm. Thereafter they will be seen by a physician at 6 and 12 months and will have nurse contact at 2, 4, 8, 12, 16, 20, 30, 36, and 42 weeks. Again, any changes to medication will be made using a standardized staged protocol (see Medical management).

Using goal-oriented motivational interviewing techniques, the aim of the physical activity intervention will be to encourage patients to safely increase and maintain their exercise level to at least 150 minutes per week, and aiming for 240 minutes per week of vigorous intensity exercise per week. This goal will be applied to all participants, regardless of their initial level of physical activity. The activity goal will be achieved gradually over 12 weeks in a step-wise fashion and then maintained for the remainder of the study (see Table 5). During the first week, participants will simply be encouraged to do something active on three to four days per week. This will be based outside the hospital setting and monitored as outlined below. On subsequent weeks, the activity level will be increased to 85, 100, 110, 115, 130 and finally 150 minutes per week. The intensity level will also gradually increase until the patient is performing at an intensity of 75% of their estimated maximum oxygen uptake. If participants do not achieve the physical activity goal within 12 weeks, they will be encouraged to achieve it as soon as possible thereafter. Participants will be encouraged and supported to achieve this goal, and will be instructed that they may accumulate activity throughout each day in bouts of at least 10 minutes. Participants who are active sporadically (for example, seasonally) will be encouraged to achieve the goal consistently throughout every month of the study.

**Table 5:** Graded exercise protocol

**Week**	**Total minutes per week**	**Intensity (% VO2max)**	Heart rate target (bpm)*	**RPE Borg scale**
**1**	75	55	(220-age × intensity %)^a^	
**2**	85	60		Light exercise
**3 and 4**	100	60		
**5 and 6**	110	65		
**7**	115	70		Moderate exercise
**8 and 9**	115	70		
**10 and 11**	130	75		Heavy breathing / sweating
**12 onwards**	150 (minimum)	75		

This exercise program is designed to encourage a gradual increase in physical activity over the course of 12 weeks. The starting point on the scale depends on the individual’s current fitness level.

From 12 weeks onwards, the aim is to achieve a minimum of 150 minutes per week of vigorous-intensity exercise.

RPE Borg Scale: Ratings of Perceived Exertion on a scale of 6 to 20; Heart rate in bpm (beats per minute*), ^a^for example, a 45 year-old man wishing to undertake exercise at 65% intensity would aim for a heart rate of (220 to 245) × 0.65 = 114 bpm [37].

All participants will be instructed to monitor their physical activity on a daily basis throughout the study using a wrist-worn heart rate monitor (Polar, Warwick, England) and physical activity log. The heart rate monitor will be worn whilst conducting exercise and will record the length of exercise and the heart rate during exercise. Participants will be requested to keep a daily log of activity and their blood glucose (BG). Participants will be asked to return the completed activity and BG logs to the research nurse at each visit. These will inform the subsequent discussion, and help monitor and encourage an increase in activity levels.

Use of the diary and heart rate monitor will be taught in the first meetings with the research nurse after randomization. Participants will negotiate appropriate targets with the nurse based initially on baseline values and subsequently on achievement. The setting of negotiated, realistic, achievable targets, and self-monitoring of progress towards these targets, is a key strategy for developing confidence to exercise (self-efficacy) and is a strategy adopted by many regular exercisers. All self-monitoring records will be reviewed by the research nurse. The activity log will be copied and returned to the participant, with written or verbal comments from the research nurse. The comments will highlight examples of positive changes the participant has made and help the participant address any barriers to physical activity encountered.

Participants who have not reached the activity goal of an extra 150 minutes per week of moderate to vigorous physical activity within four months of randomization will be offered further help to reach this target. An appointment will be made with a personal fitness instructor to examine alternative ways of helping them increase their activity. They will also be offered discounted membership at their local leisure center, where they will have access to gym and pool facilities and numerous supervised exercise classes (done through the exercise prescription). However, our previous experience indicates that such additional interventions are likely to be required in only a minority of cases.

#### Medical management

Management of T1DM, blood pressure, and lipid profile will be undertaken by the study team for the duration of the study. Any changes in treatment of these features will be made according to NICE guidelines [38] to keep the risk of performance bias to a minimum.

Glycemic control influences beta cell function, therefore our aim will be to achieve optimal glycemia in both arms of the study. To help with this, all patients will be managed either on insulin pump therapy or on a multiple dose insulin injection regime (fast acting or soluble insulin with each meal and background insulin at night). At each visit, the research nurse will review the patients blood glucose and make changes to insulin dosages if need be, and advice will be given about how to alter carbohydrate intake and insulin dosages to minimize hypoglycemic episodes with exercise and obtain ideal glucose control before, during and after exercise.

The overall aim will be to maintain the following targets: HbA1c concentration lower than 6.5%, blood pressure lower than 140/80 mmHg, total cholesterol concentration lower than 4·0 mmol/L, HDL cholesterol concentration higher than 1·0 mmol/L, LDL cholesterol concentration lower than 2·0 mmol/L, and concentration of triglycerides lower than 2·0 mmol/L.

### Assessment

**Figure 1:**
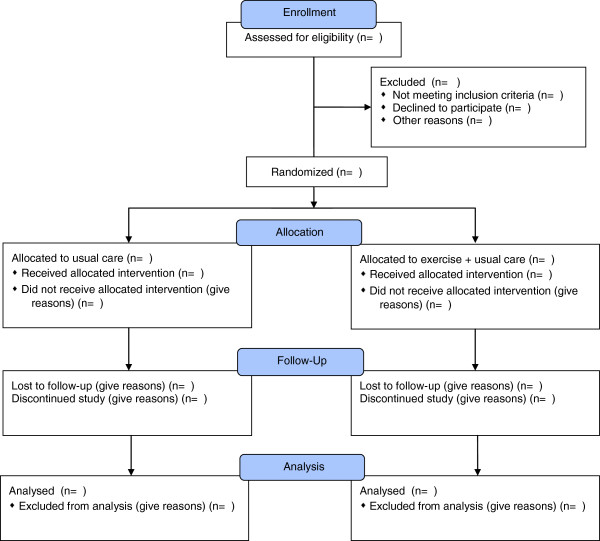
Consort diagram describing flow of patients through study.

Information will be collected on the number of patients diagnosed with T1DM, who have been approached and the reasons for not coming into the study, so that a full consort diagram can be generated (see Figure 1).

Participants will be assessed at baseline, 6 and 12 months. A schedule and details of assessments that will be carried out are given in Tables 3 and 4.

The clinical examination, and assessment of HbA1c, lipids, and renal and thyroid function constitute part of good clinical care and will inform the clinical management of the patient.

Downloads from the heart rate monitor and review of the exercise diaries will confirm and quantify the exercise being undertaken by patients in the exercise arm. Data from the activity monitors will enable us to assess whether we have managed to increase activity in both arms of the study.

Changes across the study in the C peptide response to a mixed meal will enable us to determine the effect that exercise has on beta cell protection.

Measurement of inflammatory markers will inform on the antiinflammatory effect of exercise. Measurement of islet antibodies will allow confirmation and sub-classification of T1DM for the final analysis of the data.

### Safety

Written advice on carbohydrate and insulin dose adjustment around exercise will be prepared and distributed to both health care professionals and patients. The study team will keep in contact with subjects as they start and increase their exercise participation so that the risks of hypoglycemia and injury can be minimized. Any such events will be documented and reviewed at the study meetings.

### Planned sample size and analyses

A minimum of 30 patients per arm is considered sufficient to provide meaningful data to be obtained from this pilot study, but this will be reconsidered in the light of information gathered in phase 1. An initial recruitment rate of 30% is anticipated followed by a 90% adherence rate to the exercise schedule and a 15% drop-out rate. In order to complete the pilot study with a minimum of 30 patients, this means that 30/(0.85 × 0.9 × 0.3) = 130 patients will need to be approached initially.

With the varying sample sizes described above, estimates and 95% confidence intervals on recruitment rate, exercise adherence rate and drop-out rate will be:

Recruitment rate 30% (22% to 39%)

Adherence rate 90% (76% to 97%)

Drop-out rate 15% (5% to 30%)

Summary statistics (mean, standard deviation (SD), median, interquartile range (IQR), proportions) of demographic and primary outcome variables will be presented separately for intervention and control patients. Also, separately for intervention and control patients, change from baseline to 12 months in fitness related variables listed in Tables 3 and 4 will be estimated together with 95% confidence intervals. Trends over time in fitness related variables will be illustrated graphically. Variability in these changes will also be estimated in order to be able to estimate sample size requirements for a definitive trial. Comparison between intervention and control patients in change from baseline in fitness related variables will be made giving effect size estimates for the definitive trial. Association between fitness and demographic variables will be examined at a preliminary level using correlation analysis and chi-squared. A generalized linear model (GLM) will be used to get an initial impression of significance of differences between intervention and control groups in fitness related variables. Demographic variables and baseline fitness variable levels will be included as covariates. Further analysis will use non-linear mixed model analysis for outcome related fitness variables measures including the 19 sites as a random effect. Variation from site to site will then be used to further assist in the design of the definitive trial. The effect of demographic variables will also be investigated with mixed effect modeling.

### Ethics

The study has received approval from the Birmingham East, North and Solihull Research Ethics Committee, UK and local NHS Research and Development review panels. The study will conform to The International Conference on Harmonization of good clinical practice (GCP) guidelines as well as with the Declaration of Helsinki. A steering committee has been established to monitor the trial.

## Discussion

There is increasing evidence that exercise preserves beta cell function in individuals who are overweight, glucose intolerant or have type 2 diabetes. It is not known whether this is true in people with type 1 diabetes. This pilot study aims to provide data on recruitment rates, adherence to exercise programs, drop-out rates and rate of loss of beta cell function in this group of patients. This information is required to design a formal trial of exercise to preserve beta cell function in newly diagnosed type 1 diabetes.

This study design is of a multi-center, randomized controlled trial and is therefore particularly robust. The length of intervention (one year) is longer than most other studies of exercise in type 1 diabetes. The initial qualitative interviews will provide valuable information on the barriers to exercise in patients with type 1 diabetes in the UK, to enable healthcare practitioners to target exercise advice more effectively.

One of the limitations of the study is the small sample size. Due to the incidence of type 1 diabetes and the age group we are recruiting, it is not possible to recruit large numbers of people with recently diagnosed type 1 diabetes even from multiple centers. As this is a pilot study, one of the primary outcome measures is recruitment rates. If this pilot study shows that recruitment rate is poor, we will explore recruitment from a larger number of centers nationally or internationally. A further limitation is the unsupervised nature of the intervention. This makes assessment of adherence difficult. We are using downloadable heart rate monitors and accelerometer data to validate participants’ exercise diaries.

Whilst this is the first study to look at the effects of exercise on beta cell function in type 1 diabetes, studies have examined other benefits of exercise in this condition. Exercise has been demonstrated to improve fitness, insulin requirement, lipids, insulin resistance and well- being, and to reduce cardiovascular disease and mortality in people with long standing type 1 diabetes [39].

Preservation of beta cells in new or incipient type 1 diabetes will have benefits to the patient as well as the NHS. For patients, it can help avoid late complications, and for the NHS, it can help reduce the costs associated with treating these complications. Should exercise preserve beta cell function, it would be advantageous and straightforward to enable an educational/motivational program through existing healthcare providers targeted at increasing exercise levels in patients with new type 1 diabetes.

## Trial status

Recruitment commenced in November 2011 and is expected to continue until July 2013. Open to recruitment.

## Abbreviations

GCP: Good clinical practice; GLM: Generalized linear model; HbA1c: Glycated hemoglobin; IQR: Interquartile range; RPE: Ratings of perceived exertion; SD: Standard deviation; T1DM: Type 1 diabetes mellitus; T2DM: Type 2 diabetes mellitus; VO2max: Maximal oxygen consumption

## Competing interests

The authors declare that they have no competing interests.

## Authors’ contributions

AD, GD, and SG helped prepare the protocol and provided advice on phase 1 of the study. DT and KS helped prepare the protocol and provided advice on exercise aspects of phase 2 of the study. RH and colleagues in the Primary Care Clinical Trials Unit provided statistical advice. NL, AK, NJ, RA and PN wrote the manuscript with contribution from all co-authors. All authors read and approved the final manuscript.

## Steering committee

Dr Spiros Fourlanos, Royal Melbourne Hospital, Australia

Professor Tim Barrett, Birmingham Children’s Hospital, Birmingham, UK

Dr Ian Gallen, Buckinghamshire Healthcare NHS Trust, UK.

## Study website

http://www.birmingham.ac.uk/extod

## References

[B1] Atkinson MA Eisenbarth GS Type 1 diabetes: new perspectives on disease pathogenesis and treatment Lancet 2001 358 9277 221 229 10.1016/S0140-6736(01)05415-0 11476858

[B2] Gardner SG Bingley PJ Sawtell PA Weeks S Gale EA Rising incidence of insulin dependent diabetes in children aged under five years in the Oxford region: time trend analysis. The Bart’s-Oxford study group BMJ 1997 315 7110 713 717 10.1136/bmj.315.7110.713 9314756 PMC2127500

[B3] Making every young person with diabetes matter. Report of the children and young people with diabetes working group 2007 London: Department of Health Diabetes Policy Team

[B4] Sherry NA Tsai EB Herold KC Natural history of beta cell function in type 1 diabetes Diabetes 2005 54 Suppl 2 S32 S39 16306337 10.2337/diabetes.54.suppl_2.s32

[B5] Scholin A Islet antibodies and remaining beta cell function eight years after diagnosis of diabetes in young adults: a prospective follow-up of the nationwide diabetes incidence study in Sweden J Intern Med 2004 255 3 384 391 10.1046/j.1365-2796.2003.01273.x 14871463

[B6] Greenbaum CJ Preservation of beta cell function in autoantibody positive youth with diabetes Diabetes Care 2009 32 10 1839 1844 10.2337/dc08-2326 19587365 PMC2752937

[B7] Keenan HA Sun JK Levine J Residual insulin production and pancreatic beta cell turnover after 50 years of diabetes: Joslin medalist study Diabetes 2010 59 2846 2853 10.2337/db10-0676 20699420 PMC2963543

[B8] Montanya E Fernandez-Castaner M Soler J Improved metabolic control preserved beta cell function two years after diagnosis of insulin dependent diabetes mellitus Diabetes Metab 1997 23 4 314 319 9342545

[B9] The DCCT Research Group Effect of intensive therapy on residual ß cell function in patients with type I diabetes in the diabetes control and complications trial Ann Intern Med 1998 128 517 523 9518395 10.7326/0003-4819-128-7-199804010-00001

[B10] Sjoberg S Residual insulin production, glycemic control and prevalence of microvascular lesions and polyneuropathy in long-term type 1 (insulin-dependent) diabetes mellitus Diabetologia 1987 30 4 208 213 10.1007/BF00270417 3297896

[B11] Steffes MW Beta cell function and the development of diabetes-related complications in the diabetes control and complications trial Diabetes Care 2003 26 3 832 836 10.2337/diacare.26.3.832 12610045

[B12] Fleming A What will it take to get therapies approved for type 1 diabetes? Ann N Y Acad Sci 2008 1150 25 31 10.1196/annals.1447.043 19120263

[B13] Kruszynska YT Basal and 24-hour C peptide and insulin secretion rate in normal man Diabetologia 1987 30 1 16 21 10.1007/BF01788901 3552817

[B14] Ludvigsson J GAD treatment and insulin secretion in recent-onset type 1 diabetes N Engl J Med 2008 359 18 1909 1920 10.1056/NEJMoa0804328 18843118

[B15] Little RR Rohlfing CL Tennill AL Standardization of C peptide measurements Clin Chem 2008 54 1023 1026 10.1373/clinchem.2007.101287 18420730

[B16] Wang L Lovejoy N Faustman DL Persistence of prolonged C peptide production in type 1 diabetes with an ultrasensitive C peptide assay Diabetes Care 2012 35 465 470 10.2337/dc11-1236 22355018 PMC3322715

[B17] Greenbaum CJ Dead or alive? Diabetes Care 2012 35 3 459 460 10.2337/dc11-2441 22355015 PMC3322728

[B18] Thrower SL James L Hall W Green KM Arif S Allen JS Van-Krinks C Lozanoska-Ochser B Marquesini L Brown S Wong FS Dayan CM Peakman M Proinsulin peptide immunotherapy in type 1 diabetes: report of a first-in-man phase I safety study Clin Exp Immunol 2009 155 2 156 165 10.1111/j.1365-2249.2008.03814.x 19040615 PMC2675245

[B19] Herold KC Gitelman SE Willi SM Gottlieb PA Waldron-Lynch F Devine L Sherr J Rosenthal SM Adi S Jalaludin MY Michels AW Dziura J Bluestone JA Teplizumab treatment may improve C peptide responses in participants with type 1 diabetes after the new-onset period: a randomized controlled trial Diabetologia 2013 56 2 391 400 10.1007/s00125-012-2753-4 23086558 PMC3537871

[B20] Chatenoud L Immune therapy for type 1 diabetes mellitus - what is unique about anti-CD3 antibodies? Nat Rev Endocrinol 2010 6 3 149 157 10.1038/nrendo.2009.275 20173776

[B21] Choi SB Jang JS Park S Estrogen and exercise may enhance beta cell function and mass via insulin receptor substrate 2 induction in ovariectomized diabetic rats Endocrinology 2005 146 11 4786 4794 10.1210/en.2004-1653 16037383

[B22] Choi SB Jang JS Hong SM Jun DW Park S Exercise and dexamethasone oppositely modulate beta cell function and survival via independent pathways in 90% pancreatectomized rats J Endocrinology 2006 190 2 471 482 10.1677/joe.1.06400 16899580

[B23] Coskun O Ocakci A Bayraktaroglu T Kanter M Exercise training prevents and protects streptozotocin-induced oxidative stress and beta cell damage in rat pancreas Tohoku J Exp Med 2004 203 3 145 154 10.1620/tjem.203.145 15240923

[B24] Park S Hong SM Lee JE Sung SR Exercise improves glucose homeostasis that has been impaired by a high-fat diet by potentiating pancreatic beta cell function and mass through IRS2 in diabetic rats J Appl Physiol 2007 103 5 1764 1771 10.1152/japplphysiol.00434.2007 17761790

[B25] Ertek S Cicero A Impact of physical activity on inflammation: effects on cardiovascular disease risk and other inflammatory conditions Arch Med Sci 2012 8 5 794 804 23185187 10.5114/aoms.2012.31614 PMC3506236

[B26] Dela F Von Linstow ME Mikines KJ Galbo H Physical training may enhance beta cell function in type 2 diabetes Am J Physiol Endocrinol Metab 2004 287 5 E1024 E1031 10.1152/ajpendo.00056.2004 15251867

[B27] Bloem CJ Chang AM Short-term exercise improves beta cell function and insulin resistance in older people with impaired glucose tolerance J Clin Endocrinol Metab 2008 93 387 392 10.1210/jc.2007-1734 18000089 PMC2243226

[B28] Kitabchi AE Temprosa M Knowler WC Kahn SE Fowler SE Haffner SM Andres R Saudek C Edelstein SL Arakaki R Murphy MB Shamoon H Diabetes Prevention Program Research Group Role of insulin secretion and sensitivity in the evolution of type 2 diabetes in the Diabetes Prevention Program: effects of lifestyle intervention and metformin Diabetes 2005 8 2404 2414 16046308 10.2337/diabetes.54.8.2404 PMC1360738

[B29] Slentz CA Tanner CJ Bateman LA Durheim MT Huffman KM Houmard JA Kraus WE Effects of exercise training intensity on pancreatic beta cell function Diabetes Care 2009 32 10 1807 1811 10.2337/dc09-0032 19592624 PMC2752909

[B30] Li G Zhang P Wang J Gregg EW Yang W Gong Q Li H Li H Jiang Y An Y Shuai Y Zhang B Zhang J Thompson TJ Gerzoff RB Roglic G Hu Y Bennett PH The long-term effect of lifestyle interventions to prevent diabetes in the China Da Qing diabetes prevention study: a 20-year follow-up study Lancet 2008 371 9626 1783 1789 10.1016/S0140-6736(08)60766-7 18502303

[B31] Knowler WC Barrett-Connor E Fowler SE Hamman RF Lachin JM Walker EA Nathan DM Diabetes Prevention Program Research Group Reduction in the incidence of type 2 diabetes with lifestyle intervention or metformin N Engl J Med 2002 346 6 393 403 11832527 10.1056/NEJMoa012512 PMC1370926

[B32] Andrews RC Cooper AR Montgomery AA Norcross AJ Peters TJ Sharp DJ Jackson N Fitzsimons K Bright J Coulman K England CY Gorton J McLenaghan A Paxton E Polet A Thompson C Dayan CM Diet or diet plus physical activity versus usual care in patients with newly diagnosed type 2 diabetes: the Early ACTID randomized controlled trial Lancet 2011 378 9786 129 139 10.1016/S0140-6736(11)60442-X 21705068

[B33] Chief Medical Officers of England, Scotland, Wales, and Northern Ireland Helath D Start active, stay active: a report on physical activity from the four home countries’ Chief Medical Officers 11 June 2011 UK: Department of Health, Physical activity, Health improvement and protection

[B34] American Diabetes Association Standards of medical care in diabetes - 2012 Diabetes Care 2012 35 S11 S63 22187469 10.2337/dc12-s011 PMC3632172

[B35] Greenhalgh T Taylor R Papers that go beyond numbers (qualitative research) BMJ 1997 315 740 743 10.1136/bmj.315.7110.740 9314762 PMC2127518

[B36] ACSM’s guidelines for exercise testing and prescription 2010 Eighth Lippincott: Williams and Wilkins 76 79

[B37] Swain DP Abernathy KS Smith CS Lee SJ Bunn SA Target heart rates for the development of cardiorespiratory fitness Med Sci Sport Exer 1994 26 1 112 116 8133731

[B38] National Institute for Clinical Excellence Type 1 diabetes: diagnosis and management of type 1 diabetes in children, young people and adults [internet] 2012 [updated 2010 March]. Available from: http://www.nice.org.uk/CG15

[B39] Chimen M Kennedy A Nirantharakumar K Pang TT Andrews R Narendran P What are the health benefits of physical activity in type 1 diabetes mellitus? A literature review Diabetologia 2012 55 3 542 551 10.1007/s00125-011-2403-2 22189486

